# Zoology, chemical composition, pharmacology, quality control and future perspective of Musk (Moschus): a review

**DOI:** 10.1186/s13020-021-00457-8

**Published:** 2021-06-19

**Authors:** Kai Liu, Long Xie, Mao Deng, Xumin Zhang, Jia Luo, Xiaofang Li

**Affiliations:** School of Pharmacy, Chengdu University of Traditional Chinese Medicine, Chengdu, 611137 People’s Republic of China

**Keywords:** Musk, Muscone, Pharmacology, Quality control

## Abstract

Musk, the dried secretion from the musk sac gland which is located between the navel and genitals of mature male musk deer, is utilized as oriental medicine in east Asia. It has been utilized to treat conditions such as stroke, coma, neurasthenia, convulsions, and heart diseases in China since ancient times. This paper aims to provide a comprehensive overview of musk in zoology, chemical composition, pharmacology, clinical applications, and quality control according to the up-to-date literature. Studies found that musk mainly contains macrocyclic ketones, pyridine, steroids, fatty acids, amino acids, peptides, and proteins, whilst the main active ingredient is muscone. Modern pharmacological studies have proven that musk possesses potent anti-inflammatory effects, neuroprotective effects, anti-cancer effects, antioxidant effects, etc. Moreover, muscone, the main active ingredient, possesses anti-inflammatory, neuroprotective, antioxidant, and other pharmacological effects. In the quality control of musk, muscone is usually the main detection indicator, and the common analytical method is GC, and researchers have established novel and convenient methods such as HPLC-RI, RP-UPLC-ELSD, and Single-Sweep Polarography. In addition, quality evaluation methods based on steroids and the bioactivity of musk have been established. As for the identification of musk, due to various objective factors such as the availability of synthetic Muscone, it is not sufficient to rely on muscone alone as an identification index. To date, some novel technologies have also been introduced into the identification of musk, such as the electronic nose and DNA barcoding technology. In future research, more in vivo experiments and clinical studies are encouraged to fully explain the pharmacological effects and toxicity of musk, and more comprehensive methods are needed to evaluate and control the quality of musk.
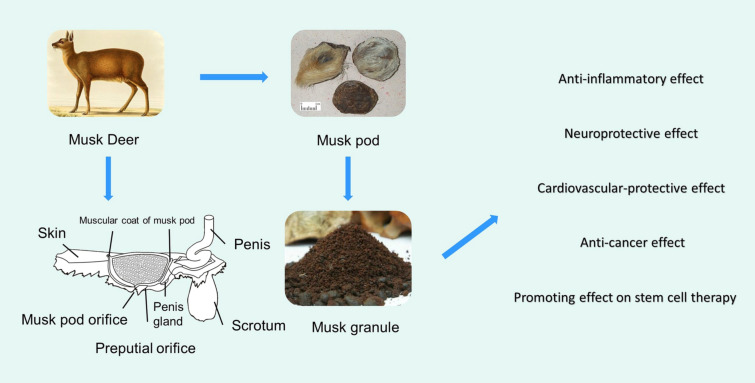

## Introduction

Natural musk is the dried secretion from the musk sac gland which located between the navel and genitals of mature male *Moschus berezovskii* Flerov (Forest musk deer), *Moschus sifanicus* Przewalski (Alpine musk deer), or *Moschus moschiferus* Linnaeus (Siberian musk deer) of the Cervidae family [1–3]. Natural musk is initially recorded in Shen Nong’s Classic of the Materia Medica (Shen Nong Ben Cao Jing). It possesses the efficacy of opening the orifices (resuscitating), invigorating blood and promoting menstruation, relieving swelling and pain. Meanwhile, it has been utilized as a kind of medicine to treat stroke, coma, neurasthenia, convulsions, heart diseases, ulcerous sores, and other conditions for 2000 years in China [2, 4, 5]. Because of its potential efficacy, musk is often used in combination with other traditional Chinese medicines to treat diseases. For example, Xihuang Wan, containing Bovis calculus, Olibanum, Myrrha, and Moschus, is a traditional prescription for clearing heat and detoxifying, reducing phlegm and resolving masses, promoting blood circulation and eliminating swelling, as well as removing stasis and relieving pain. It is mainly used to treat breast cancer, buboes, scrofula, subcutaneous nodule, deep multiple abscesses, pulmonary abscess, and small intestinal abscesses [1]. In addition to being used for medicinal purposes, natural musk has been used in the perfume industry for hundreds of years in Europe, due to the low output and wide application of natural musk, it cost five times as much as gold once in Europe, and now is prohibitively so [6, 7].

**Fig. 1:**
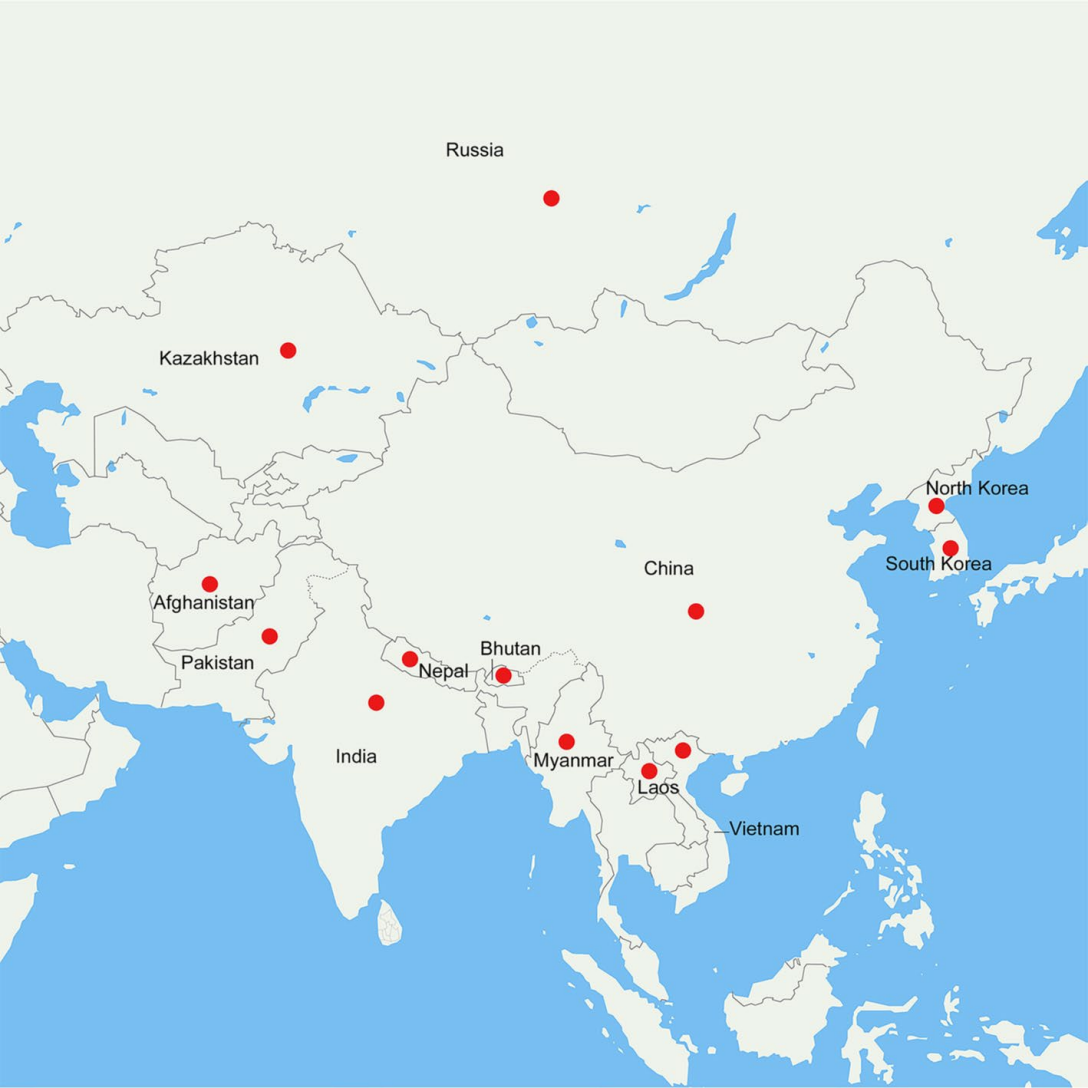
Countries where musk deer are distributed

As the source of the natural musk, geographically, musk deer are mainly distributed across at least 13 countries in Asia (Fig. 1). To date, seven species have been discovered in the aggregate worldwide, while the specified sources of natural musk in Chinese Pharmacopoeia (2020 edition) are *Moschus berezovskii* Flerov, *Moschus sifanicus* Przewalski, and *Moschus moschiferus* Linnaeus [1, 8]. Traditionally, people had to kill musk deer to obtain musk in the past, which eventually led to a steep decline in the population of musk deer in the past 3–4 decades [9]. One study estimated that the musk deer population in China was no more than 0.1 million by the end of the last century, while that in the 1950s was 2.5 million [10]. According to data from the International Union for Conservation of Nature, six out of the seven species are endangered [8, 10, 11]. Moreover, the population of 7 species of musk deer is still decreasing [6, 12]. Accordingly, they are currently listed in Appendix I in the Convention on International Trade in Endangered Species of Wild Fauna and Flora and Category I of the State Key Protected Wildlife List of China [8, 13]. To ensure the sustainable use of natural musk, the Chinese Government stipulated that only 4 Chinese patent medicine are allowed to use natural musk during preparation, namely Angong Niuhuang Pill, Liushen Pill, Babao Dan, and Pien Tze Huang. Also, the group led by the Institute of Materia Medica Chinese Academy of Medical Science developed artificial musk, a musk-like mixture mainly containing synthetic muscone and other substitutes, in 1993 in China in response to the shortage of natural musk [14]. Moreover, the group won the first prize of the National Science and Technology Progress Award in 2015. However, the specific details are not known since the method of manufacturing artificial musk is a state secret. Modern pharmacological and biological experiments had shown that artificial musk has similar activities and indications as natural musk [2, 15]. Meanwhile, farming became a vital way to protect musk deer and the only legal way to obtain the natural musk. The farming of musk deer started in 1958 and preserving the wild populations at the same time in China, and expansion of musk deer farming has been made from then on [3, 16].

In this paper, the zoology, chemical composition, pharmacological properties, toxicity, pharmacokinetics, and quality control of musk are reviewed. Relevant information about musk and musk deer was collected from the website about Big Data of Traditional Chinese Medicine, the Official website of an international organization. Relevant literature on musk was collected from scientific databases including PubMed, ScienceDirect, Web of Science, Springer, Wiley, and CNKI, spanning the years 1906–2020. The purpose of this review is to summarize the relevant information of musk with emphasis on its pharmacological activities and quality control, so as to provide more up-to-date information and inspiration for future research.

## Zoology

**Fig. 2:**
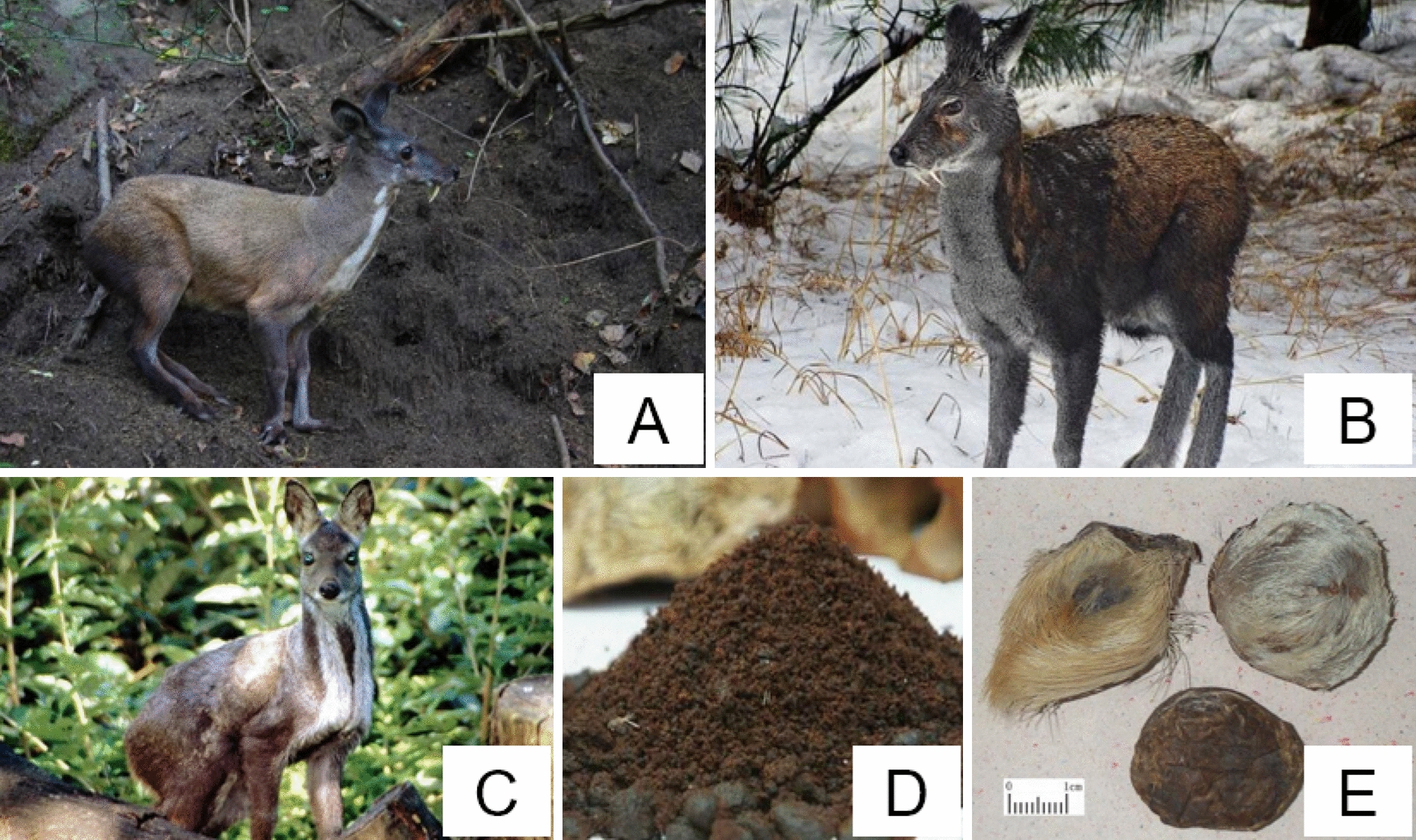
Musk deer and musk. **A** *Moschus berezovskii* Flerov, **B** *Moschus sifanicus* Przewalski, **C**. *Moschus moschiferus* Linnaeus, **D**. Musk, E. Musk sachets

The musk deer is a kind of protected and economical animal in China (Fig. 2). Alpine musk deer body hair is sandy brown, the rear is brown. Its body hair is tan and the hair on the back end of the ear is brown. Of the three animals that are sources of musk, the forest musk is the smallest, they weigh 7 to 9 kg and are 70 to 80 cm long, followed by the Siberian musk deer (9–13 kg and 70–90 cm long) and then the Alpine musk deer (10–15 kg and 80–90 cm long). Male ones of the three species possess well-developed canines that expose outside the lips. The canines of the Alpine musk deer are wider than those of the Forest musk. Their snouts are not the same length, the snout of Forest musk deer is short, but the snout of Alpine musk deer is longer. Forest musk deer is similar in shape and hair color to Siberian musk deer. Its hair color is gray-brown or dark brown and darker than that of Siberian musk deer and Alpine musk deer, meanwhile, the hair color on its hip is much deeper and the stripes under its neck are obvious. The hair on the back end of the ear is brown and that on the base of the ear and within the auricle is white or yellowish-white. There is no spot on the back of the mature male Forest musk deer. Adult Alpine deer has 4–6 large brown patches on the back of the neck, with a few fuzzy spots on the rear. The hair on the jaw is white and stripes under the neck are light yellow or off-white. Adults of Siberian musk deer distributed in northeastern China and the Dabie Mountains in Anhui have cinnamon-colored spots. The hair color of deer distributed in the Qinling Mountains and west of Sichuan is darker and without spots. The stripes around the neck are obvious, and there are spots on that of cubs. The tails of all three species of musk deer are short and hidden in the fur [17, 18].

Musk obtained from wild musk deer is soft, oily, and loose. The surfaces of irregular spherical or granular ones are mostly purple-black, oily, and shiny, with a few lines, and the section is dark brown or yellow–brown. The powdery ones are mostly tan or yellow–brown, and consist of a small amount of fine hair and shed inner membrane. Musk obtained from domestic musk deer is granules, short strips, or irregular clumps. The surface of these clumps is uneven, purple-black or dark brown, oily, slightly shiny, with a small amount of hair and shed inner membrane. Musk possesses an intense and peculiar aroma and tastes slightly spicy, slightly bitter, and salty [1].

## Chemical composition

**Fig. 3:**
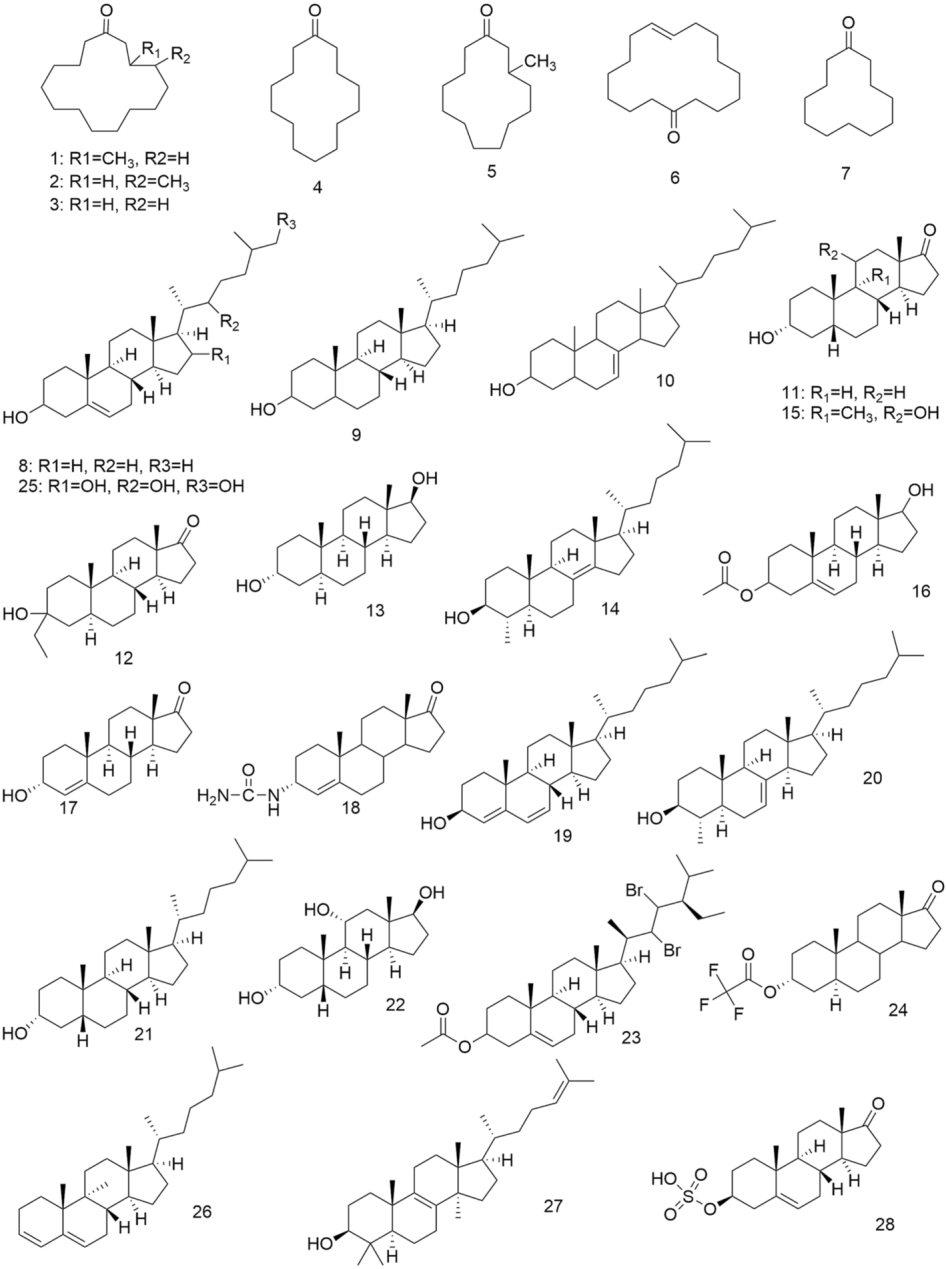
Chemical structures of some cyclic ketones and steroids in musk

Forest musk deer is the most widely distributed and most farmed species in China. In addition, after a literature search, it was found that researchers have studied the chemical composition of forest musk the most, therefore, this section will discuss forest musk. The composition of natural musk is complex and variable [19]. It mainly contains macrocyclic ketones, pyridine, steroids, fatty acids, amino acids, peptides, and proteins [2, 19–21]. Moreover, the active ingredients in musk are mainly macrocyclic ketones, steroids, and some peptides. Some chemical structures of active components in musk are shown in Fig. 3.

### Macrocyclic ketones

Muscone (3-methylcyclopentadecan-1-one) (**1**), one of the macrocyclic ketones, was isolated by Walbaum in 1906 and characterized by Ruzicka et al. in 1926 [22–24]. After decades of research, it is considered as the major medicinal active and odor-contributing ingredient of natural musk [21, 22, 25–27]. Moreover, 4-methylcyclopentadecan-1-one (**2**), normuscone (cyclopentadecanone) (**3**), cyclotetradecanone (**4**), 3-methylcyclotridecan-1-one (**5**), cyclohexadec-8-en-1-one (**6**), cyclododecanone (**7**) have been isolated from musk [21].

### Steroids

Steroids in musk are variable and they are the second-largest lipid component in musk, and these compounds contain many androstane derivatives, with which the androgenic effects of musk are closely linked [19–21]. Some steroids have been isolated from musk thus far, such as Cholesterol (**8**), Cholestan-3-ol (**9**), Cholest-7-en-3β-ol (**10**), 3α-hydroxy-5β-androstan-17-one (**11**), 3-ethyl-3-hydroxy-5α-androstan-17-one (**12**), 5α-androstane-3α,17β-diol (**13**), 4α-methyl-5α-cholest-8(14)-en-3β-ol (**14**), 3,11-dihydroxy-(3α,5β,11α)-androstan-17-one (**15**), 3-acetate, (3β,17β)-androst-5-ene-3,17-diol (**16**), 3α-hydroxyandrost-4-en-17-one (**17**), 3α-ureido-androst-4-en-17-one (**18**), 4,6-cholestadien-3β-ol (**19**), 4α-methyl-5α-cholest-7-en-3β-ol (**20**), 5β-cholestan-3α-ol (**21**), 5β-androstan-3α,11α,17β-triol (**22**), 22,23-Dibromostigmasterol acetate (**23**), Androsterone, trifluoroacetate (**24**), Cholest-5-ene-3,16,22,26-tetrol (**25**), Cholesta-3,5-diene (**26**), lanosterol (**27**), and dehydroepiandrosterone sulfate (**28**) [21].

## Pharmacological effects

**Fig. 4:**
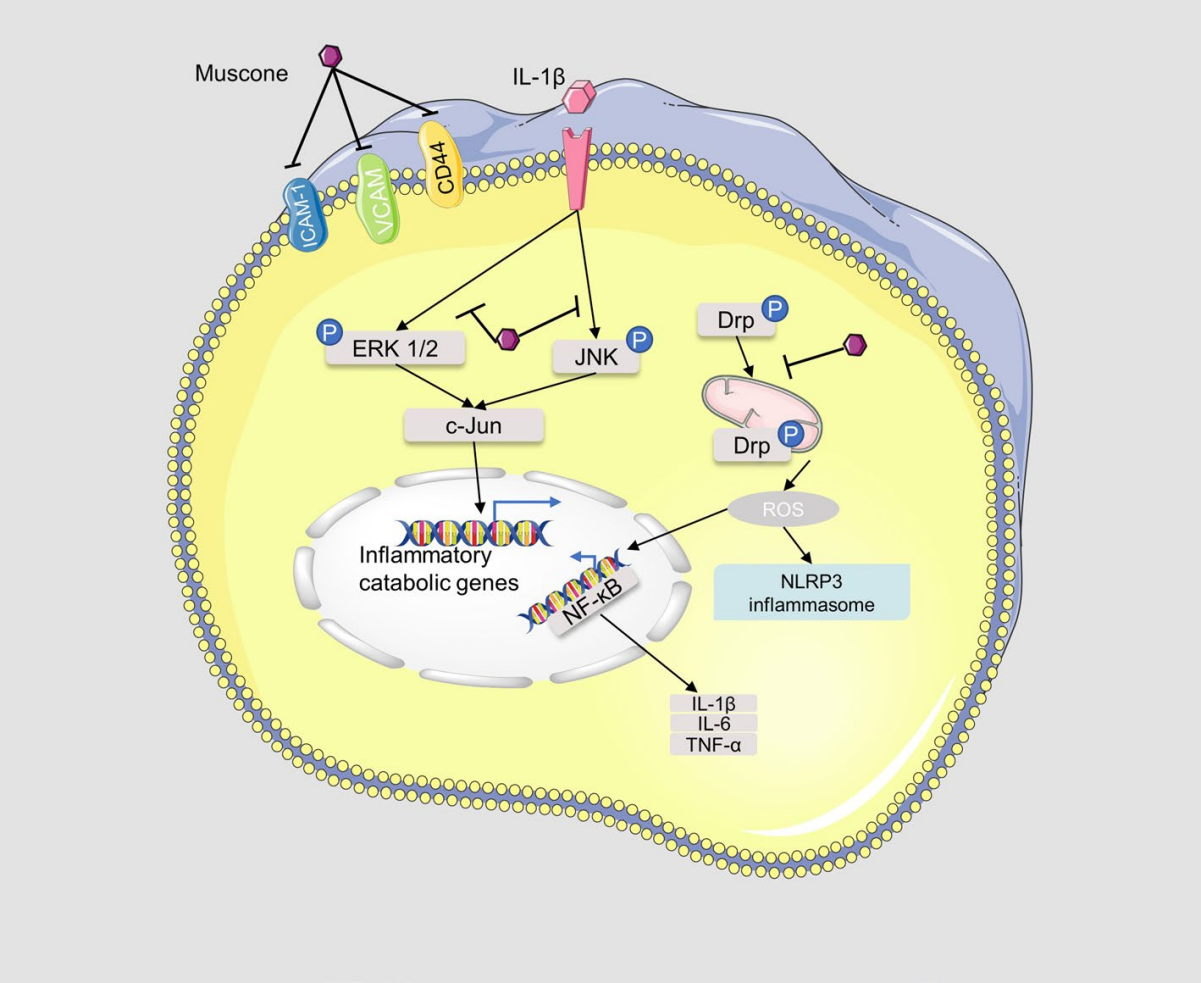
The mechanisms of musk against inflammatory diseases

**Fig. 5:**
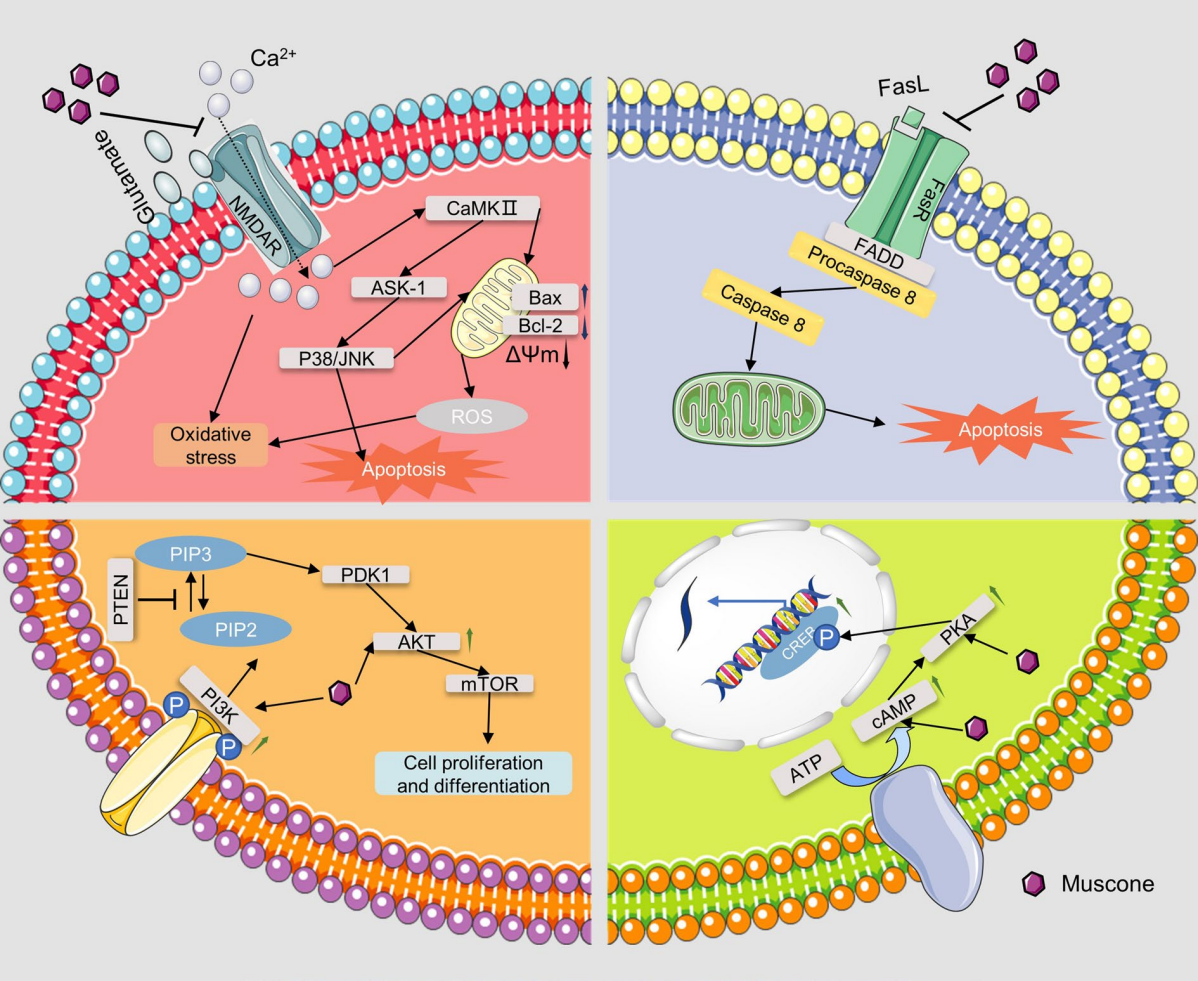
The neuroprotective effect of musk

**Fig. 6:**
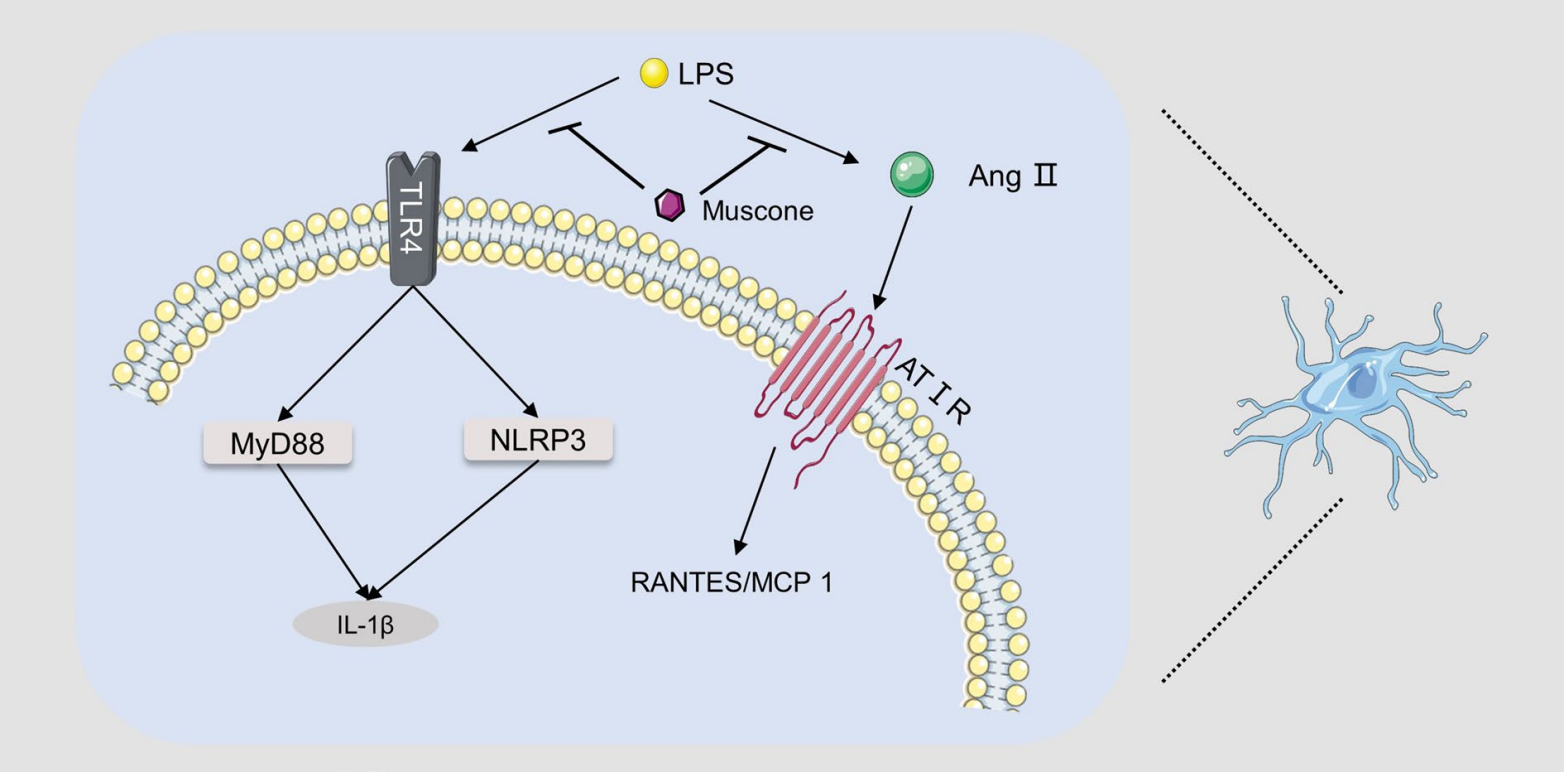
The protective effect of musk against microglial cell inflammation

According to relevant literature, musk and its main active ingredient, muscone, possess various pharmacological effects such as anti-inflammatory activity, neuroprotective activity, and cardiovascular-protective activity. All the specific details are shown in Table 1 and some relevant molecular mechanisms are depicted in Figs. 4, 5, 6.

**Table 1:** Pharmacological activities of musk

	Models	Active extract /compounds	Concentration /doses	Activity/potential mechanism	Refs.
Anti-inflammatory activity	Rat neutrophils	Glycoprotein musk-1	1, 10, 100 μg/mL	Slightly increasing PLA2 activity, significantly inhibiting ALOX5 activity, and significantly increasing COX activity	[41]
	Rat neutrophils	Glycoprotein musk-1	1, 10, 100 μg/mL	Inhibiting the release of lysosomal enzymes	[42–45]
	Rat neutrophils	Glycoprotein musk-1	1, 10, 100 μg/mL	Inhibiting the production of neutrophil platelet-activating factor and the activity of acetyl-CoA-dependent acetyltransferase	[46]
	Rat neutrophils	Glycoprotein musk-1	1, 10, 100 μg/mL	Significantly inhibiting the chemotaxis of neutrophils	[47]
	Carrageenin edema; Formalin arthritis; Adjuvant arthritis	Musk	0.5, 1.0, 5.0 mg/100 g, i.p.; 1.0, 5.0 mg/100 g, i.p.; 1.0, 5.0 mg/100 g, i.p	Reducing the histamine and S-HT contents of the inflamed tissues	[34]
	HUVEC	Muscone	37.5, 75, 150 μg/mL	Decreasing expression of CAMs on HUVEC	[49]
	IL-1β induced end-plate chondrocytes; Rat model of endplate degeneration	Muscone	6.25, 12.5, and 25 lmol/L; 10 mg/kg, p.o	Blocking the proinflammatory effect of IL-1β in vitro; inhibiting inflammatory cytokine expression in degenerated IVDs, and prevent IVD degeneration in vivo	[52]
	Murine BV2 microglial cells; adjuvant inflammatory pain model	Muscone	1, 2, 4, 8, 16 μM; 4, 8, 16 mg/kg	Suppressing microglial activation-mediated inflammatory response through the NOX4/JAK2-STAT3 pathway and NLRP3 inflammasome; inhibiting the CFA-induced NOX4, p-JAK2/p-STAT3, and NLRP3 inflammasome expression in the spinal cord of mice	[54]
Neuroprotective effects	Glutamate-induced PC12 cells	Muscone	0.1, 1, 10 μM	Attenuating ROS generation and Ca2 + influx, via NR1 and CaMKII-depended ASK-1/JNK/p38 signaling pathways	[61]
	MCAO rat model	Muscone	1 mg/kg, i.g	Down-regulating the expression of EAAC1mRNA in the ischemic hippocampus	[62]
	MCAO rat model	Muscone	1 mg/kg, i.g	Reducing NR1 protein expression, thereby reducing excitatory glutamate toxicity	[63]
	MCAO rat model; oxygen–glucose deprivation cell model	Muscone	0.5, 1 mg/kg, i.g.; 0.9, 1.8 μM	Activating the PI3K/Akt signaling pathway	[67]
	In vitro blood–brain barrier model	Muscone	8 μM	Inhibiting P-gp and MMP-9 expression	[59]
	The traumatic brain injury model	Muscone	1.8 mg/kg, nasal administration	reducing the water content of brain tissue, alleviating cerebral edema, promoting secretion of BDNF and NGF by olfactory ensheathing cells	[71]
	Traumatic brain injury rat model	Muscone	1.8 mg/kg, intranasal administration	Reducing cerebral edema and activating the PKA-CREB signal pathway	[72]
	Complete cerebral ischemia/reperfusion rat model	Muscone	0.9, 1.8, 3.6 mg/kg, i.g	Reducing oxidative stress damage, delaying neuronal death effects, and inhibiting excitotoxicity caused by EAA	[64]
	Pentylenetetrazol induced rat epilepsy model	Muscone	10, 50, 100 mg/kg, i.p	Inhibiting the c-Fos, c-jun expression	[73, 74]
	PC12 cells; MCAO rat model	Muscone	100, 300 ng/mL; 0.08, 0.16 mg	Inhibiting apoptosis and Fas pathway	[66]
	LPS-treated mice	Muscone	2 mg/kg, i.p	Repressing neuroinflammation in the prefrontal cortex of mice caused by its suppression on microglia activation and production of inflammatory cytokines via acting on TLR4 pathway and RAS cascade	[25]
Cardiovascular-protective effects	H_2_O_2_ induced H9c2 cardiomyocytes; H_2_O_2_ induced HUVEC	Musk	50 μg/mL	Scavenging ROS and improving the activity of intracellular antioxidant enzymes	[78, 79]
	H_2_O_2_ induced HUVEC	Muscone	3, 15, 30 μg/mL	Stabilizing cell mitochondrial membrane potential, reducing cell permeability, and preventing Ca2^+^ influx	[80]
	Myocardial infarction rat model	Muscone	2 mg/kg, i.g	Reducing the expression of transforming growth factor-β1, TNF-α, IL-1β and NF-κB	[82]
	Myocardial infarction rat model	Muscone	2 mg/kg, i.g	Stimulating angiogenesis via upregulating HIF-1α and VEGFA	[83]
	Myocardial infarction mice model	Muscone	2 mg/kg, i.g	Inhibiting NF-κB and NLRP3 inflammasome activation, thereby improving cardiac function	[84]
	Neonatal rat cardiac myocytes	Muscone	0.215, 0.43 or 0.86 μg/mL	Alleviating the increase of lactic acid dehydrogenase release, malondialdehyde production, creatine kinase activity, caspase-3 activity, [Ca2 +]i, apoptosis rate and expression of Bax protein, and reduction of superoxide dismutase activity, MMP, and expression of Bcl-2 protein	[85]
Anti-cancer effects	Breast cancer mice model	Muscone	2 mg/kg, i.g	Reducing the expression of VEGF	[88]
	HepG2 cells; xenograft liver cancer model	Muscone	0.663 μM; 0.1 mmol/kg, 0.2 ml/20 g bw	Increasing cellular apoptosis through endoplasmic reticulum stress responses and inducing autophagy through AMP kinase/mTOR complex 1 signaling pathway	[89]
Promoting effect on stem cell therapy	Human GMSCs	Muscone	3, 6, 9 mg/L	Increasing the proliferation and migration, promoting the adipogenic differentiation and inhibiting the osteogenic differentiation of GMSCs by inhibiting the Wnt/β-catenin signaling pathway	[93]
	Skull bone defect rat model	Musk	42, 86, 168 mg/kg	Promoting stromal cell-derived factor 1 and monocyte chemotactic protein 1 expression to promote the migration of exogenous bone marrow mesenchymal stem cells to bone injury sites in rats	[97, 98]
	Skull bone defect rat model	Musk	42 mg/kg	Increasing the level of SDF-1, HGF, and SCF, and expression of MCP-1 mRNA, FGF-2 mRNA, TGF-β mRNA, VEGF mRNA, and down-regulating the expression of EGF mRNA	[99–102]
	Human GMSCs; alcohol-induced osteonecrosis of the femoral head	Muscone	1, 10, 25 μM; 1 mg/kg	Promoting ALP activity and mRNA expression of COL1 and OCN; restoring BV/TV ratio and bone density of necrotic femoral heads	[103]
	Gentamicin-induced AKI	Muscone	3.0 mg/L	Up-regulating the expression of CXCR4 and CXCR7	[104]

### Anti-inflammatory effects

Inflammation is a kind of biological function triggered by the rupture of mechanical tissue or the reaction caused by physical, chemical, or biological agents in the body. The diseases associated with inflammation include cardiovascular disease, arthritis, cancer, diabetes, Alzheimer's disease, Parkinson's disease, etc. [30]. Studies have shown that the anti-inflammatory effect of traditional Chinese medicine (TCM) is achieved by inhibiting the expression of master transcription factors, pro-inflammatory cytokines, chemokines, intercellular adhesion molecules, and pro-inflammatory mediators [31]. Modern studies have proven that natural musk is an anti-inflammatory agent [32, 33] and some molecular mechanisms are depicted in Fig. 4.

Subcutaneous injection of musk Tween 80 emulsion could reduce croton oil-induced inflammatory response in male albino rats [32]. Taneja et al. [34] investigated the inhibitory effect and mechanism of musk on acute and chronic inflammation models, including the carrageenan-induced edema and formalin arthritis model. The mechanism study indicated that the anti-inflammatory effect of musk may be related to the reduction of histamine and 5-hydroxytryptamine (5-HT) content in inflammatory tissues. Another study also showed that musk has antihistamine and anti-5-HT effects [35]. Moreover, the aqueous extract of musk residues that have been extracted with diethyl ether and 95% ethyl alcohol and a polypeptide (musk-1) with a molecular weight less than 10,000 Da in this extract have also attracted great interest from researchers [36, 37]. In the early stage, Zhu et al. found that intravenous administration of the aqueous extract and musk-1 counteracted effectively croton oil-induced ear inflammation in mice, respectively [37]. Further studies demonstrated that this aqueous extract was effective in a variety of inflammatory models. In addition, the anti-inflammatory effect of intravenous musk-1 on croton oil-induced ear inflammation in mice was 36 times greater than that of hydrocortisone [38]. Moreover, it indicated that musk could modulate the immune function of the body and the presence of adrenal glands is necessary for the anti-inflammatory effect of musk [38, 39]. Mechanism studies indicated that the aqueous extract could inhibit platelet aggregation and arachidonic acid metabolism pathway, increase cyclic adenosine monophosphate levels [39, 40]. Subsequently, Wang et al. conducted a series of experiments to study the anti-inflammatory mechanism of musk-1 using rat neutrophils as subjects. The results showed that musk-1 could inhibit 5-lipoxygenase activity in neutrophils [41], the release of lysozyme [42–45], and platelet-activating factor production and acetyl-CoA-dependent acetyltransferase activity [46]. Meanwhile, musk-1 could significantly inhibit the chemotactic response of neutrophils [47]. These effects may be important mechanisms for their anti-inflammatory effects.

Moreover, muscone was proved to possesses anti-inflammatory effects and utilized to treat inflammatory disorders. Excessive inflammation can lead to slow wound healing [48]. He et al. [49] investigated the regulatory mechanism of muscone on chronic wound inflammation. Muscone was found to significantly inhibit the expression of ICAM-1, VCAM-1, and CD44 on the surface of human umbilical vein endothelial cells (HUVEC), thereby inhibiting the adhesion of polymorphonuclear leukocytes to HUVEC to suppress excessive inflammation and promote healing of chronic wounds. Interleukin (IL)-1 initiates and controls the inflammatory response [50]. Studies demonstrated that overproduction of IL-1β played an important role in the pathogenesis of human intervertebral disc degeneration [51]. Liang et al. [52] studied the protective effect of muscone on vertebral end-plate degeneration. In vitro, muscone inhibited the IL-1β-induced phosphorylation of extracellular signal-regulated kinases 1/2 and c-Jun N-terminal kinase in a dose-dependent manner. In vivo, muscone inhibited the expression of prostaglandin E2, 6-keto-prostaglandin F1a, IL-1β, and tumor necrosis factor α and recovered the structural distortion of the degenerative disc. Moreover, the therapeutic potential of muscone on cervical spondylotic myelopathy (CSM) was evaluated by Zhou et al. In the chronic cervical cord compression rat model, muscone promoted motor recovery in rats. Molecular studies revealed that muscone could inhibit activation of the NLRP3 inflammasome, NF-κB, and Drp1 in lesions to attenuate inflammatory responses and neuronal damage in model rats [53]. In LPS-stimulated BV2 and primary microglial cells, muscone could inhibit the NLRP3 inflammasome and NF-κB activation to suppress mRNA levels of IL-1β, IL-6, and TNF-α and iNOS and Cox-2 protein expression [53]. These results indicated that the potential of muscone to treat CSM is due in part to its anti-neuroinflammatory effects. In addition, intraperitoneal injection of muscone could reduce inflammatory pain by blocking the NOX4/JAK2-STAT3 pathway and NLRP3 inflammasome, which can cause an inflammatory response [54].

### Neuroprotective effects

Natural musk has the action of inducing resuscitation and has been utilized as a TCM in treating stroke, coma, neurasthenia, convulsions for thousands of years [4]. Modern studies have demonstrated the neuroprotective effects of musk. Ayuob et al. found that in a depression model, inhaling musk could improve behavioral changes, elevated serum glucocorticoid levels, memory impairment, neurodegenerative changes, and changes in salivary gland structure induced by chronic unpredictable mild stress [55–58].

In addition, the neuroprotective effects of muscone were evaluated by researchers (Figs. 5 and 6). Wang et al. demonstrated that muscone could change the permeability of the blood–brain barrier (BBB) model in vitro. The mechanism is related to reducing the expression of permeability glycoprotein (P-gp) and matrix metallopeptidase 9 (MMP-9) [59]. This may be one of the reasons why Muscone could cross the BBB to reach the lesion sitePlease do not break the line before the next sentence. Muscone has a therapeutic effect on cerebral ischemia. In vitro studies showed that muscone could inhibit glutamate-induced PC12 cell apoptosis [60]. A further mechanistic study suggested that this effect was attributed to muscone reducing reactive oxygen species (ROS) production and Ca^2+^ influx through NR1 and camki-dependent ASK-1/JNK/p38 signaling pathway [61]. Liang et al. found that in the MCAO rat model, muscone could effectively down-regulate the expression of EAAC1 mRNA to achieve its neuroprotective effect [62]. Moreover, the mechanism may also be related to reducing NR1 protein expression [63]. In addition, Sun et al. [64] demonstrated that muscone had an obvious neuroprotective effect on mice with complete cerebral ischemia. This protective effect attributed to the fact that muscone can increase the superoxide dismutase (SOD) content of the brain tissue of rats, reduce the malondialdehyde (MDA) content, reduce the increase of excitatory amino acids (EAA) content caused by ischemia and hypoxia, and inhibit the excitatory neurotoxicity caused by EAA. Fas is a death receptor and is of paramount relevance in stroke pathogenesis [65]. It is suggested that neutralizing FasL would be a great choice for stroke treatment. In cerebral ischemia rats, muscone exerted a neuroprotective effect through inhibiting apoptosis by suppressing the expression of Fas [66]. Post-stroke recovery is also important for patients and neural stem cells (NSCs) are of importance in this process. Muscone can promote neural stem cell proliferation and differentiation to protect against cerebral ischemia. This effect is attributed to the activation of the PI3K/Akt signaling pathway [67]. Cerebral ischemia is accompanied by edema, and this symptom may lead to death [68]. Muscone can alleviate edema of brain tissue in the ischemic area and significantly reduce the brain water content to play a protective role. In addition, muscone can also change the permeability of the BBB, reduce albumin exposure and leakage, and reduce the degree of edema of brain cells [69]. Jiang et al. [70] found that in the early period after traumatic brain injury, muscone could exert neuroprotective effects by inhibiting the expression of MMP-9 and reducing cerebral edema.

Moreover, muscone exerts protective effects against traumatic brain injury (TBI). Intranasal administration of muscone can promote the secretion of brain-derived neurotrophic factor and nerve growth factor by olfactory ensheathing cells to exert a neuroprotective effect [71]. Another study demonstrated that muscone exerted neuroprotective effects after TBI by activating the PKA-CREB signal pathway [72]. Cheng et al. studied the mechanism of anti-epilepsy activity of muscone and found that muscone blocked the expression of c-Fos and c-Jun in the brain during seizures, and this effect had a dose–effect relationship [73, 74]. Recently, He et al. proved that muscone could improve depression-like behavior in rats by repressing lipopolysaccharide (LPS)-induced neuroinflammation. The underlying mechanism may be its suppression of microglia activation and production of IL-1β through acting on TLR4/MyD88 and TLR4/NLRP3 as well as its blockade on the expression of RANTES and MCP-1 (monocyte chemotactic protein 1) via antagonizing renin/Ang II axis [25].

Taken together, the above findings suggest that musk has good neuroprotective effects and has great potential for treating neurological diseases. Some related molecular mechanisms are depicted in Figs. 5 and 6, and the details are summarized in Table 1.

### Cardiovascular-protective effects

Cardiovascular disease is the deadliest disease worldwide, and its morbidity and mortality rates continue to rise. Studies have shown that some herbs or active ingredients in them have the potential to treat cardiovascular diseases, such as curcumin [75], baicalin [76], and berberine [77]. There is evidence that musk is also effective against cardiovascular disease. Quan et al. found that musk can play a protective role against H_2_O_2_-induced H9C2 cardiomyocytes injury by eliminating ROS and improving intracellular antioxidant enzyme activity [78]. Moreover, musk can play a protective role in H_2_O_2_-induced HUVEC injury by improving intracellular antioxidant enzyme activity and reducing oxidative stress [79]. Also, researchers investigated the effect of muscone on cardiovascular disease in vitro and in vivo. Hong et al. demonstrated that muscone can stabilize mitochondrial ΔΨm, reduce cell permeability and reduce Ca^2+^ influx, thereby inhibiting HUVEC cell apoptosis induced by H_2_O_2_ [80]. Zhou et al. [81] studied the application of muscone in random skin flap transplantation. Muscone can promote skin flap angiogenesis, activate VEGF expression, reduce apoptosis, increase SOD levels and decrease MDA levels. Therefore, muscone can improve the survival rate of skin flaps by anti-oxidation, anti-apoptosis, and promoting angiogenesis. Moreover, myocardial infarction (MI) is the leading cause of death and disability in developed countries, and a number of challenges remain in preventing and treating MI. Wang et al. found that muscone could improve cardiac remodeling and dysfunction caused by MI. The mechanistic study revealed that muscone could reduce the expression of transforming growth factor-β1 (TGF-β1), tumor necrosis factor-α (TNF-α), IL-1β, and nuclear factor-κB (NF-κB) to reduce the inflammatory response. Moreover, muscone could reduce myocardial apoptosis by upregulating the Bcl-2/Bax ratio. What’s more, the intervention of muscone significantly induced the phosphorylation of Akt and eNOS, which is related to vascular endothelial function [82]. Further, Du et al. demonstrated that muscone improved cardiac function in mice with MI by enhancing angiogenesis. The underlying mechanism of this effect was up-regulating hypoxia-inducible factor 1α (HIF-1α) and vascular endothelial growth factor A (VEGFA) expression levels [83]. Similarly, by reducing macrophage-mediated chronic inflammation, muscone can improve cardiac function in mice with MI. The mechanism was to inhibit the activation of NF-κB and NLRP3 inflammasome, thereby blocking the production of inflammatory cytokines (IL-1β, TNF-α, and IL-6) [84]. When cardiomyocytes were pretreated with muscone before I/R injury, the increase of LDH release, MDA production, creatine kinase activity, caspase-3 activity, [Ca^2+^]_i_, apoptosis rate and expression of Bax protein, and reduction of SOD activity, MMP, and expression of Bcl-2 protein can be alleviated. This suggested that muscone can protect I/R injury by inhibiting cellular oxidative stress and apoptosis [85].

### Anti-cancer effects

Musk is widely used to treat cancer. It is included in many traditional Chinese medicine formulae for treating cancer, such as the Xihuang pill [86]. Xu et al. [87] studied the effects of musk and muscone on 22 types of tumor cells. It was found that musk and muscone could widely induce cancer cell growth inhibition and apoptosis. In a nude mouse model of blood stasis syndrome, muscone can significantly inhibit the growth of breast cancer. The mechanism may be related to the reduction of VEGF expression [88]. Qi et al. [89] found that muscone had a certain anti-cancer effect in hepatocellular carcinoma and this effect attributed to the induction of apoptosis and autophagy of liver cancer cells. The mechanistic study showed that apoptosis was a consequence of endoplasmic reticulum stress through the PERK/ATF4/DDIT3 signaling pathway, and autophagy was closely related to the AMP kinase/mTOR complex 1 signaling pathway. P-gp is a product of the multidrug resistance (MDR) gene, and the high expression of P-gp on the tumor cell membrane is the main mechanism of MDR formation [90]. Wang et al. used human colon carcinoma cell line Caco-2 as a target and proved that muscone can effectively inhibit the function of P-gp [91].

### Promoting effect on stem cell therapy

Nowadays, mesenchymal stem cells (MSCs) are widely used in stem cell therapy [92]. Related reports have demonstrated that musk has the effect of promoting mesenchymal stem cell therapy and the details are summarized in Table 1. In vitro, muscone (3, 6, 9 mg/L) can enhance the proliferation of human gingival mesenchymal stem cells (GMSCs), and 6 mg/L of muscone had the best effect. In vivo, muscone can effectively inhibit osteoblast differentiation and promote GMSC proliferation, migration, and adipogenesis, which is attributed to the inhibition of the Wnt/β-catenin signaling pathway [93]. In a skull defect rat model, muscone (4.2, 8.4, 16.8 μL/100 g) could promote the migration of exogenous stem cells in vivo, and the effect was better at concentrations of 4.2 and 8.4 μL/100 g [94], and the mechanism was related to the promotion of BMSCs proliferation and osteogenic differentiation and the promotion of exogenous BMSCs migration in vivo [95, 96]. Studies have shown that the mechanism by which musk promoted the migration of exogenous bone marrow mesenchymal stem cells to the injury site may be related to its promotion of MCP-1 expression and SDF-1 (stromal cell-derived factor-1) expression in bone defects [97, 98]. Li et al. investigated the mechanism by which musk promotes the healing of bone defects in the skull of rats. The mechanism of musk promoted healing may be related to the increase of serum SDF-1 and hepatocyte growth factor (HGF) levels, the up-regulation of mRNA expression of stem cell factor (SCF), MCP-1, fibroblast growth factor 2 (FGF-2), TGF-β, and VEGF, as well as the down-regulation of mRNA expression of epidermal growth factor (EGF) [99–102]. Guo et al. found that muscone had a protective effect on femoral head necrosis caused by alcohol. In vitro, muscone had the potential to promote alkaline phosphatase (ALP) activity and mRNA expression of collagen 1 (COL1) and osteocalcin (OCN) in ethanol-treated hBMSCs. In vivo, muscone could restore BV/TV ratio and bone density of necrotic femoral heads [103]. In addition, in an acute kidney injury (AKI) model, muscone enhanced the therapeutic effect of bone marrow mesenchymal stem cells by promoting cell proliferation, secretion, and migration. The mechanism may be related to the expression of C-X-C chemokine receptor (CXCR) 4 and 7 up-regulation [104].

### Other effects

In addition to the pharmacological effects mentioned above, other pharmacological effects of musk and muscone have also been reported, including inducing liver drug metabolism enzymes, antibacterial, etc. Muscone can induce certain P-450 isoenzymes, which in turn can alter the metabolism and endogenous substrates of drugs. Pretreatment with muscone (75 mg/kg) for 1 day can increase 2.8 times of benzophenantamine demethylase activity in rat microsomes [105]. Tanaka et al. studied the effect of muscone on rat liver microsomal drug metabolism enzyme system and other enzyme activity parameters in vitro and in vivo and found that muscone could induce liver metabolism enzymes [106]. Muscone mainly induced P450 IIB1 and P450 IIB2 with a slightly weaker effect than phenobarbital [107]. Recently, Phung et al. studied the preventive effect of muscone against cisplatin nephrotoxicity. In LLC-PK1 cells, muscone was proved to prevent cisplatin-induced oxidative stress, inflammation, and apoptosis. The mechanistic studies revealed that muscone could inhibit ROS accumulation and induce HO-1 expression to exert an antioxidant effect in cisplatin-treated LC-PK1 cells. Meanwhile, muscone could suppress the phosphorylation of p38, which may mediate production of TNF-α. Moreover, in cisplatin-treated LC-PK1 cells, muscone played an anti-apoptotic role by inhibiting p53, caspase-3, 7, and 8, and restoring the Bcl-2/Bax ratio [108]. In addition, the protective role of muscone in postmenopausal osteoporosis was evaluated by Zhai et al. [109] employing bone marrow monocytes, RAW264.7, and female C57BL/6 ovariectomized mice. In vitro, muscone inhibited osteoclastogenesis in BMMs and RAW264.7 cells. In vivo, the bone loss was prevented by muscone by suppressing osteoclastogenesis. The over-activated RANKL signaling pathways will promote the reproduction of osteoclasts. The molecular study demonstrated that muscone could reduce the levels of RANK and TRAF6, leading to the suppression of downstream NF-kB and MAPK signaling pathways.

Musk extract had inhibitory and bactericidal effects on the growth of pathogenic bacteria such as *Staphylococcus aureus* and *Penicillium* [110]. Saddiq studied the inhibitory effects of musk on five opportunistic pathogenic fungi, namely *Aspergillus flavus*, *Aspergillus fumigates*, *Rhizopus stolonifer*, *Fusarium solani*, and *Candida albicans*. Musk extract (25%) had an inhibitory effect on the above fungi, the inhibition rates were 74.61%, 68.76, 56.92%, 71.57%, and 67.80%, respectively. Subsequent animal experiments showed that musk extract can reduce lung toxicity caused by *A. flavus* [111]. AL-Jobori et al. studied the antifungal activity of musk in vitro. Five kinds of fungi were used, including *Aspergillus fumigates*, *Aspergillus niger*, *Alternaria *Spp*.*, *Trichomphyton mentagrophytes*, and *Fusarium *Spp. All concentrations (25, 50, 75, or 100%) and amounts (1, 2, 4 mL) inhibited fungal growth and completely eliminated the fungi [112]. Meanwhile, musk also inhibited the activity of hydatid cyst [113]. Dong et al. demonstrated that muscone (0.1, 1, 10, 50 mol/L) could reduce high glucose-induced autophagy and apoptosis in RSC 96 cells, and its mechanism was to activate the AKT/mTOR signaling pathway [26].

## Clinical application

Musk possesses a wide range of pharmacological effects. In modern clinical applications, musk and muscone are utilized to treat diseases and they are mostly used in combination with other Chinese herbal medicines. To date, many clinical trials have been listed in the global clinical trial registry (https://clinicaltrials.gov) and the Chinese Clinical Trial Registry (http://www.chictr.org.cn).

Internationally, a total of 8 clinical trials related to musk have been registered, among which four are related to musk Shexiang Baoxin Pill (NCT01897805, NCT03072121, NCT04026724, NCT04022031), one is related to Mayinglong musk hemorrhoid ointment (NCT01881282), one is related to Gongjin-dan (NCT03219515), one is related to Qishe Pill (NCT01274936), and one is related to Angong Niuhuang Pill (NCT00817609). For example, the therapeutic effect and safety of compound carraghenates cream with Mayinglong musk hemorrhoid ointment in the treatment of hemorrhoids, especially regarding the relief of pain. The curative effect of Shexiang Baoxin Pill on coronary artery disease not amenable to revascularization based on western medicine therapy was evaluated. Of these, one trial has been completed (NCT01881282), five trials are of unknown status, and two observational trials related to Shexiang Baoxin Pill have not yet enrolled patients. But unfortunately, none of these clinical trials have published results.

In China, 15 clinical trials of Chinese patent medicines containing musk have been registered since 2012. These tests are mostly related to Shexiang Tongxin Dropping Pill (ChiCTR2000035167, ChiCTR2000032429, ChiCTR1900025810, ChiCTR-IPC-17010823, ChiCTR-IPR-16009785, ChiCTR-IPR-16008950, ChiCTR-IPR-15006020,) and Shexiang Baoxin Pill (ChiCTR2000041451, ChiCTR2000034817, ChiCTR1900027946, ChiCTR-TRC-10001237, ChiCTR-TRC-12003513). Moreover, all of these clinical trials are related to cardiovascular disease. For example, the Second Affiliated Hospital of the Second Military Medical University studied the therapeutic effect of Shexiang Tongxin Dropping Pill on myocardial perfusion among acute myocardial infarct patients (Shexiang Tongxin Dropping Pill). Recently, a clinical trial of Shexiang Baoxin Pill in the treatment of coronary microvascular dysfunction has been prospectively registered (ChiCTR2000034817). Furthermore, a trail of musk used to treat acute ST-elevation myocardial infarction has also been in preparation (ChiCTR2000037470).

Overall, clinical trials related to musk or Chinese patent containing musk are gradually increasing, especially in China, which is of great significance for more scientific and full utilization of musk.

## Toxicity and safety

The related report indicated that muscone had toxic effects on zebrafish (AB = type) embryo development. Muscone (5, 10, 20, 40, 80, 100 μmol/L) had a lethal effect on zebrafish embryos. When the concentration of muscone reached 80 and 100 μmol/L, the embryo death rate reached 100% at 96 h after fertilization. High-dose muscone had a significant effect on zebrafish embryo development in a time- and dose-dependent manner, which mainly manifested as abnormal development of muscle tissue and heart tissue [114]. Muscone (0.005, 0.01, 0.03, 0.1, 0.2 mM) was toxic to zebrafish embryos by increasing Myh6 and Myh7 mRNA expression and reducing thyroid genes (Trh, Thrβ, and Dio3) expression [115]. Muscone could induce CYP1A2 and CYP3A4 expression in liver cells in vitro and in vivo. In addition, when the dose exceeded 50 mg/kg, muscone had significant liver toxicity in Kunming mice [116]. Further, pharmacodynamic drug-drug interactions (DDIs) occur when the pharmacological effect of one drug is altered by that of another drug in a combination regimen [117]. Liu et al. [118] demonstrated that muscone would reduce the hypnotic and analgesic effects of ketamine, a widely used general anesthetics, in a dose-independent manner, which may be related to changes in NR1 and delta-opioid receptors. Hence, when a patient is given muscone preoperatively, it is important to monitor the depth of anesthesia during the surgery.

## Pharmacokinetics

The pharmacological activity of a drug in the body is closely related to its absorption, distribution, metabolism, and excretion process in the body. In view of the extensive usage of musk in TCM, an in-depth study of the pharmacokinetics of it is necessary. Unfortunately, the pharmacokinetics of musk are poorly studied globally. On the other hand, there are some pharmacokinetics studies on the muscone, the main active component. At the early stage, Zhu et al. established a method employing gas chromatography and applied it to the determination of blood concentration after oral administration of muscone. After oral administration of 80 mg/kg muscone, the parameters indicated that the whole blood concentration–time curve of muscone in rats was best fitted to a two-compartment open model. The T_1/2_Ka (min), T_max_ (min), C_max_ (mg/L) and T_1/2_β (min) were 22, 74.4, 1.44 and 196.1, respectively. These results indicated that muscone was absorbed quickly and eliminated quickly in rats, [119]. After that, Zhu et al. utilized the same method to determine the pharmacokinetic parameters of intravenously administered muscone in rats, rabbits, and dogs. After intravenous administration of muscone (12, 18 and 24 mg/kg) to rats, the T_1/2_α, T_1/2_β, V_ss_ and V_c_ were 9.4–9.6 min, 118.1–131.2 min, 22.5–23.5 L/kg and 2.3–2.9 L/kg, respectively. The whole blood concentration–time curve was best fitted to a two-compartment open model. Whilst AUC (μg·min^−1^/mL) were 153.0, 207.7 and 258.2, respectively, which were dose-proportional. After intravenous administration of muscone to rabbits and dogs at a dose of 24 and 18 mg/kg respectively, the whole blood concentration–time curves were both fitted to a three-compartment open model. In rabbits, the T_1/2_α (min), T_1/2_β (min), T_1/2_γ (min), V_ss_ (L/kg) and V_c_ (L/kg) were 4.82 ± 2.60, 24.87 ± 13.62, 331.92 ± 61.32, 51.65 ± 25.61, 2.13 ± 0.84. In dogs, the T_1/2_α (min), T_1/2_β (min), T_1/2_γ (min), V_ss_ (L/kg) and V_c_ (L/kg) were 2.78 ± 3.8, 29.98 ± 22.11, 366.39 ± 185.44, 7.25 ± 2.23, 0.38 ± 0.30 [120]. Moreover, Li et al. also demonstrated that muscone can be quickly absorbed in the gastrointestinal tract, and the highest concentration of plasma and brain tissue was reached 1.5 h after intragastric administration, indicating that muscone quickly entered the brain tissue through the BBB. The elimination rate constants of muscone in brain tissue and plasma were 0.56 h^−1^ and 0.45 h^−1^, respectively, indicating that muscone was eliminated rapidly in the brain and plasma (the concentration in the brain decreases slightly faster than that in the plasma). Therefore, there was no accumulation of muscone in the brain [121].

## Quality control

As we all know, the quality difference of traditional Chinese medicine is universal. Taking musk as an example, its quality depends on the physique of the musk deer, harvest time, drying method, etc. In addition, there were reports in the early years that there was counterfeit musk on the market. Therefore, it is indispensable to establish a potentially reliable, sensitive, accurate and repeatable analysis method to ensure the quality of musk. The methods mentioned in this section are listed in Table 2.

**Table 2:** Quality control and identification methods for musk

	Methods	Conditions	Indicator/Activity	Refs.
Quality control	GC	Mobile phase, nitrogen; Column, OV-17 column; Detector, FID	Muscone	[1]
	GC/MS	Mobile Phase, helium; Column, DB-5 column; Detector, mass detector	Muscone	[122]
	HPLC-RI	Mobile phase, acetonitrile–water (95:5); Column, Zorbax ODS column; Detector, RI detector	Muscone	[123]
	RP-UPLC-ELSD	Mobile phase, methanol–water (78: 22); Column, Waters Acquity BEH C18 column; Detector, ELSD	Muscone	[124]
	Single-Sweep Polarography	Solution, phenylhydrazine hydrochloride-sodium chloride mixed aqueous solution; peak potential, -800 mV(vs.SCE)	Muscone	[125]
	GC/MS	Mobile phase, helium; Column, Rtx-5sil MS column; Detector, mass detector	Steroids	[128]
	HPLC	Mobile Phase, n-hexane-dioxane- ethyl acetate (100:2.5:0.4); Column, Self-loading silicone column (YWG_80_-5 μm); Detector, SPD-1 UV–Vis detector	Steroids	[126]
	GC/MS	Mobile phase, helium; Column, HP-1MS column; Detector, mass detector	Steroids	[127]
	Thrombin titration	Titration substrate, 0.5% bovine fibrinogen; Enzyme, 10U/mL bovine thrombin; Titration interval, 1 min; Titrant volume, 2 μL	Anti-thrombin activity	[129]
Identification	Microscopy	Microscope, Nikon E200	Physical characteristics	[130]
	GC/MS	Mobile phase, helium; Column, HP-1MS column; Detector, mass detector	Steroids	[128]
	FTIR	Nicolet 6700 FTIR spectrometer, DTGS mid-infrared detector; Dispersion medium, KBr; Spectral resolution, 4 cm^−1^; Signal accumulation, 32	Characteristic infrared absorption	[131]
	ELISA	-	MP-1	[132]
	Electronic nose coupled with chemometrics	An oxide sensor-based electronic nose (A oxide sensor-based electronic nose); musk samples, 0.03 g; Carrier gas, synthetic dry air, 150 mL/min; Injection volume, 1 mL; Injection rate, 1 mL/s, 35 °C	Odors	[133]
	DNA barcoding	DNA extraction, DNeasy tissue blood DNA Extraction Kit, modified CTAB method	Phylogenetic tree	[134]

### Quantitative quality control of musk

According to Chinese Pharmacopoeia (2020 edition), in addition to morphological and microscopic identification, as well as loss on drying and ash check, the concentration of muscone should exceed 2.0% as determined by GC [1] to control the quality of natural musk. Moreover, other methods have been established to detect muscone, such as GC–MS [122], HPLC-RI [123], RP-UPLC-ELSD [124], Single-Sweep Polarography [125]. However, the chemical composition of traditional Chinese medicine is complex, and with the advent of synthetic muscone, it is not appropriate to rely solely on muscone as an indicator of biological activity. In view of the fact that steroids are another feature in musk, some methods for the determination of steroid content in musk have been established [126–128]. Moreover, Luo et al. developed a biological evaluation method to evaluate the clinical efficacy of musk based on the biological potency of its anti-thrombin activity [129].

### Qualitative quality control of musk

Natural musk has been a precious Chinese herbal medicine since ancient times, and it has been expensive and in short supply for a long time, and hence, this situation stimulated the musk forgery. As synthetic muscone becomes available, new methods of counterfeiting may emerge. Meanwhile, the composition of natural musk is complex. Hence, it is of vital importance to seek reasonable and effective ways to identify and comprehensively evaluate natural musk. Some researchers have established methods for authenticating and evaluating natural musk. Traditionally, microscopic authentication can be used as a fast on-site method [130]. Zhang et al. used GC–MS spectrometry and searched the NIST standard library to quickly determine most of the chemical components in the musk samples, making it easier to screen the fake musk. They found that the types and content of low-content steroids were quite different and had strong characteristics. Therefore, this study focused on the analysis of the steroidal component in the samples collected. Their data showed that the steroids contained in musk were very complex and variable. However, the analysis from multiple samples can capture its characteristic components as the basis for identification. The components included in androgen hormones had strong characteristics and regularity. The establishment of fingerprints of steroid hormones can simplify data processing [128]. In addition, Zhou et al. utilized Fourier transform infrared spectroscopy (FTIR) which was fast, sensitive, intuitive, and non-destructive, to identify the authenticity of musk [131]. Ahn et al. established a direct enzyme-linked immunosorbent assay (ELISA) to identify and evaluate different sources of musk for the first time. Firstly, they purified musk protein 1 (MP-1), a unique protein, from musk and made polyclonal antibodies in rabbits. And then a direct ELISA for quantitative analysis was developed using anti-MP-1 polyclonal antibodies. Lastly, the ELISA was validated by the determination of the quantity of MP-1. MP-1was detected in four out of nine musk samples, and the concentrations that can be detected ranged from a few nanograms in 1 g of protein. The results demonstrated that this method is useful for evaluating the authenticity of natural musk [132]. The odor is an important property of natural musk and with the development of electronic nose (E-Nose), identification methods of TCM based on E-Nose are emerging. Ye et al. employed an E-Nose (αFOX-4000) to analyze the aroma of several musk samples, namely 1 artificial musk sample, 5 natural musk samples, and 3 fake musk samples. The data showed that the chemical information between different samples was severely damaged, leading to complex and fuzzy results of musk quality evaluation. Then the original data obtained from the response values of 18 sensors were analyzed by principal component analysis. The adulterates were not only easily discriminated from authentic musk samples based on the above analysis but also showed a clear separation of different quality proportions of adulterated musk [133]. Importantly, DNA barcoding has become a new direction for biological species identification and has attracted the attention of many experts. Zhao et al. designed a pair of musk mini-DNA barcode identification primers of about 180 bp and successfully identified the fake products [134].

## Discussions and future perspectives

The present review summarizes the zoology, chemical composition, pharmacological effects, toxicity, pharmacokinetics, and quality control of musk by referring to published reports. Musk is a kind of animal secretion and so far, researchers have identified macrocyclic ketones, pyridine, steroids, fatty acids, amino acids, peptides, and proteins from musk. Pharmacological studies have shown that musk has various pharmacological activities, including anti-inflammatory effects, neuroprotective effects, cardiovascular protective effects, anti-cancer effects, promoting effects on stem cell therapy, etc. Although the progress in recent decades strongly proves the medicinal value of musk, there are still some notable scientific gaps in the subsequent research.

First, the chemical composition of musk is complex. Many studies now focus only on the biological activity of muscone, ignoring the biological activity of other chemical components. However, studies have shown that muscone is not the only active ingredient in musk. For instance, the androgenic effects of musk are closely related to the androgen derivatives it contained, and decades ago scholars isolated a peptide whose anti-inflammatory activity was 20 times that of hydrocortisone [36]. As a TCM, the pharmacological effects of musk are the result of all the components working together. Therefore, it is necessary for future research to focus more on the biological activities of other components. Moreover, in pharmacological research, one problem is that many mechanisms of action have not been studied. In addition, there are many traditional uses of musk that have not been proven by modern pharmacological experiments. Furthermore, most of the current pharmacological studies have only conducted animal or in vitro studies, resulting in a lack of clinical trial data, therefore, researchers should try to convert experimental research into clinical research.

Second, there is insufficient research on the toxicity and safety of the active substances contained in musk, although it has been utilized for treating diseases for thousands of years in China. Toxicity evaluation is indispensable before conducting clinical trials and developing new drugs. Therefore, research in this area should attract sufficient attention because there are few relevant reports. Moreover, DDIs may occur when two (or more) drugs are administered together. This effect may be synergistic (enhanced potency), antagonistic (reduced potency), or the appearance of a completely new effect that does not occur when taken alone. A study suggested that muscone may affect the anesthetic effect of ketamine [118]. Meanwhile, musk is usually used in combination with other traditional Chinese medicines in practical use. Therefore, more DDIs about musk or its active substances with other drugs need to be studied.

Third, the pharmacokinetic behavior of musk needs further study. Pharmacokinetics explains how a drug is absorbed and diffused by the body after administration, the chemical changes that occur in the body, and the way the drug works and is excreted. According to the literature, there is a lack of data on the metabolism and excretion of musk in vivo. Therefore, more studies on the pharmacokinetics of musk in vivo should be conducted.

Fourth, quality evaluation of natural musk is the basis for ensuring the quality and safety of it, so it is of utmost importance to establish more complete quality control methods and standards. It is not only difficult to fully reflect the pharmacological activity and quality of natural musk by prescribing the content of muscone as the only index but also does not meet the overall viewpoint of clinical medicine for TCM. Therefore, it is necessary to improve the existing statutory quality standards. In addition, it is necessary to explore other more holistic quality control methods. There have been studies using DNA-barcoding for the quality evaluation of musk [134, 135]. The results demonstrate that this method is a promising one for the quality control method of natural musk, but more relative studies need to be done to develop this approach more comprehensively. In addition, hyperspectral imaging is also emerging in the quality control of TCM [136]. Moreover, there are seven species of musk deer, and the Chinese Pharmacopoeia (2020 edition) stipulates that three of them are natural sources of musk. Since the source of musk used in clinical practice is not uniform, and therefore its biological activity may vary, more research should be conducted on the effects of the three musks identified in the regulations.

## Conclusion

In the present review, we covered zoology, chemical composition, pharmacological effects, toxicity, pharmacokinetics, and quality control of musk as well as the zoology of musk deer. Currently, plenty of pharmacological effects of musk and its main active ingredient, muscone, have been proved by modern pharmacological research, such as anti-inflammatory effects and neuroprotection, but many other pharmacological effects related to traditional applications have yet to be proven. Simultaneously, other active substances in musk remain to be discovered and studied. Besides, there may be counterfeiting of musk in China due to the imbalance between supply and demand as well as substantial profits, yet the quantitative standards prescribed by the Chinese Pharmacopoeia (2020 edition) may not be able to fully reflect the comprehensive quality of musk. Therefore, it is of urgency to establish novel, comprehensive, and convenient musk quality evaluation methods.

## Data Availability

Not applicable.

## Abbreviations

GC: Gas chromatography
HPLC: High-performance liquid chromatography
UPLC: Ultra-performance liquid chromatography
TCM: Traditional Chinese medicine
coA: Coenzyme A
5-HT: 5-Hydroxytryptamine
ICAM-1: Intercellular adhesion molecule 1
VCAM-1: Vascular cell adhesion protein 1
HUVEC: Human umbilical vein endothelial cells
IL-1β: Interleukin 1 beta
NLRP3: NOD-, LRR- and pyrin domain-containing protein 3
LDH: Lactic acid dehydrogenase
ROS: Reactive oxygen species
MCAO: Middle cerebral artery occlusion
P-gp: Permeability glycoprotein
MMP-9: Matrix metallopeptidase 9
SOD: Superoxide dismutase
MDA: Malondialdehyde
EAA: Excitatory amino acids
LPS: Lipopolysaccharide
RANTES: Regulated upon Activation, Normal T Cell Expressed and Presumably Secreted
MCP-1: Monocyte chemotactic protein 1
TGF-β1: Transforming growth factor-β1
TNF-α: Tumor necrosis factor-α
NF-κB: Nuclear factor-κB
HIF-1α: Hypoxia-inducible factor 1 alpha
VEGFA: Vascular endothelial growth factor A
I/R: Ischemia/reperfusion
Bax: Bcl-2-associated X
MDR: Multidrug resistance
MSCs: Mesenchymal stem cells
GMSCs: Gingival mesenchymal stem cells
BMSCs: Bone marrow stromal cells
SDF-1: Stromal cell-derived factor-1
HGF: Hepatocyte growth factor
SCF: Stem cell factor
FGF-2: Fibroblast growth factor 2
EGF: Epidermal growth factor
ALP: Alkaline phosphatase
COL1: Collagen 1
OCN: Osteocalcin
BV/TV: Trabecular bone volume fraction
AKI: Acute kidney injury
CXCR: C-X-C chemokine receptor
ELISA: Enzyme-linked immunosorbent assay
MP-1: Musk protein 1
E-Nose: Electronic nose
